# Colorectal cancer cells promote osteoclastogenesis and bone destruction through regulating EGF/ERK/CCL3 pathway

**DOI:** 10.1042/BSR20201175

**Published:** 2020-06-24

**Authors:** Gong Zi-chen, Qian Jin, Zhang Yi-na, Wang Wei, Kang Xia, Xu Wei, Wu Juan, Zheng Wei

**Affiliations:** 1 College of Medicine, Southwest Jiaotong University, North Section 1 No. 111, Second Ring Road, Chengdu 610000, China; 2 Department of Orthopedics, General Hospital of Western Theater Command, Rongdu Avenue No. 270, Chengdu 610000, China; 3 Department of Pharmacy, General Hospital of Western Theater Command, Rongdu Avenue No. 270, Chengdu 610000, China

**Keywords:** bone metastasis, CCL3, colorectal cancer, EGF, osteoclast

## Abstract

Bone metastasis of colorectal cancer (CRC) cells leads to osteolysis. Aberrant activation of osteoclasts is responsible for bone resorption in tumor. In general, bone marrow-derived monocytes (BMMs) differentiate into osteoclasts, however, how CRC cells interact with BMMs and how to regulate the differentiation is elusive. We here report that CRC cells promote bone resorption in bone metastasis. Transcriptomic profiling revealed CCL3 up-regulated in MC-38 conditional medium treated BMMs. Further investigation demonstrated that CCL3 produced by BMMs facilitated cell infusion and thus promoted the osteoclastogenesis. In addition, CRC cells derived EGF stimulated the production of CCL3 in BMMs through activation of ERK/CREB pathway. Blockage of EGF or CCL3 can efficiently attenuate the osteolysis in bone metastasis of CRC.

## Introduction

Bone metastasis can occur in several cancers, including breast, lung, bladder, colorectal cancers (CRCs) [1–3]. Although the incidence is relatively low in CRC, CRC patients with bone metastasis present with a lower average age and are frequently associated with much poorer prognosis [2,4]. Previous studies indicated bone metastasis of CRC was characterized with bone resorption, which causing pathological fractures and low quality of life, but it is still largely unknown that how bone resorption is regulated in bone metastasis of CRC [2,3].

Numerous evidences showed the imbalance of functional cells within the bone, i.e. osteoblasts and osteoclasts, is responsible for the development of bone lesions, of which aberrant osteoclast activity leads to lytic bone metastasis [5–7]. In osteoclastogenesis, bone marrow-derived monocytes/macrophages (BMMs) differentiate into early osteoclast precursors which then commit to osteoclast fate [8]. However, little is known about the effect of CRC cells on BMMs. Notably, bone marrow could be the isolated metastatic site in CRC [9,10], implying the interaction between CRC cells and BMMs may be important in bone metastasis of CRC.

In the present study, we found the expression of CCL3 in primary BMMs was enhanced after treated with MC-38 conditioned medium (thereafter called CM). Overexpression of CCL3 promoted osteoclastogenesis through enhancing BMMs infusion. Further investigation revealed CRC cells derived EGF promoted the production of CCL3 in BMMs through activation of ERK/CREB pathway but not STAT3. Furthermore, blockage of EGF or CCL3 can efficiently attenuate the osteoclastogenesis and bone destruction in bone metastasis of CRC. Our findings uncover the interactions between CRC cells and BMMs and the key role of CCL3 during osteoclastogenesis in bone metastatic microenvironment of CRC. In addition, EGF and CCL3 may become potential therapeutical targets in the treatment of bone metastasis of CRC.

## Methods

### Animal experiments

All animal experiments and procedures were approved by the Animal Ethics Committee at General hospital of Western Theater Command and were performed at General hospital of Western Theater Command. C57BL/6 male mice at 8–10 weeks old were used for experiments. Mice were housed in a room with a 12:12 h light-dark cycle and given free access to tap water. Anesthesia was performed before procedures through intraperitoneally infection of 0.5% pentobarbital sodium solution. The mice were killed by CO_2_ inhalation. All experiments conformed to the guidelines of the ethical use of animals, all efforts were made to reduce the number of animals used. For histochemistry analysis, 4–8 mice were used in each group. For isolation of primary BMMs, 2–3 mice were used for each experiment.

To establish the bone metastasis model, 200,000 of MC-38 cells were injected into tibia following standard procedures following previous method [11]. For treatment with neutralizing antibody, CCL3 neutralizing antibody (2 ug/10 μl, Abcam) or EGF (2 ug/10 μl, Abcam) neutralizing antibody were injected intratibially every 3 days, the mice in control group were injected with PBS.

### Cell isolation, culture, and treatment

Cells derived from bone marrow were rinsed out, filtered and cultured with M-CSF (50 ng/ml) for 24 h to obtain BMMs. BMMs then were cultured in α-minimal essential medium (MEM) containing 10% FBS and 1% penicillin–streptomycin solution and under the treatment of RANKL (50 ng/ml). To collect MC-38 CM, MC-38 was cultured in Dulbecco's modified Eagle's medium (DMEM) supplemented with 10% fetal bovine serum (FBS), 100 U/ml penicillin (Gibco), and 100 U/ml streptomycin (Gibco). After culturing for 4 days, supernatant was discarded and was replaced with DMEM to continue to culture for 24 h, then the supernatant was collected. In some experiments, SCH772984 (50 nmol/l, MedChemExpress), EGF neutralizing antibody (0.3 μg/ml, Abcam), or recombinant CCL3 protein (100 ng/ml, Biolegend) were added to observe the effects on the activation of ERK pathway or osteoclastogenesis, DMSO or PBS solution were used as control, respectively. For transfection, siRNA-STAT3, siRNA-CREB or scramble siRNA control (scRNA) were purchased from GenePharma (GenePharma). The siRNAs or their controls were transfected into primary BMMs through INVI DNA RNA Transfection Reagent (Invigentech) following manufactures’ procedures. The sequence for siRNAs were as below: siRNA-STAT3: (sense: 5′-CCCGCCAACAAAUUAAGAATT, antisense: 5′- UUCUUAAUUUGUUGGCGGGTT); siRNA-CREB: (sense: 5′-CUGCCACAAAUCAGAUUAATT, antisense: 5′- UUAAUCUGAUUUGUGGCAGTT).

### Transcriptomic assay

Primary BMMs were seeded in six-well plates and were treated with or without CM in the presence of RANKL (50 ng/ml) for 4 days. Total RNA was extracted. RNA-Seq assay was completed by Majorbio Bio-pharm Technology Co., Ltd (Shanghai, China). Total RNA was isolated using Trizol reagent (Invitrogen) according the manufacturer's instructions. RNA-seq transcriptome library was prepared following TruSeq™ RNA sample preparation Kit (Illumina) and sequenced with the Illumina HiSeq xten (2 × 150 bp read length). Briefly, messenger RNA was isolated according to polyA selection method by oligo (dT) beads and fragmented by fragmentation buffer firstly. Then double-stranded cDNA was synthesized using a SuperScript double-stranded cDNA synthesis kit (Invitrogen) with random hexamer primers (Illumina). PCR amplified using Phusion DNA polymerase (NEB) for 15 PCR cycles. After quantified by TBS380, paired-end RNA-seq sequencing library was sequenced with the Illumina HiSeq xten/NovaSeq 6000 sequencer (2 × 150 bp read length).

The profiling data were analyzed on the free online platform of Majorbio Cloud Platform (www.majorbio.com). To identify differential expression genes (DEGs) between two different groups, the expression level of each transcript was calculated according to the TPM method. RSEM was utilized to quantify gene abundances. EdgeR was used for differential expression analysis. GO functional enrichment and KEGG pathway analysis were carried out by Goatools.

### Histochemistry and cytochemistry analysis

Hindlimbs were removed from mice at the time of sacrifice and bones were fixed in 4% paraformaldehyde (PFA) for 4 days, then the samples were washed and decalcified in a solution of 10% EDTA for 2 weeks and embedded in paraffin and sectioned at 8 μm thickness along the coronal plate from anterior to posterior. Decalcified tibial sections were stained with tartrate resistant acid phosphatase (TRAP) staining or Safranin O and fast green or H&E staining. For cytochemistry, cells were fixed in 4% paraformaldehyde (PFA) for 15 min, then the samples were washed and blocked with 3% BSA for 1 h in 37°C. Then the cells incubated in TRITC-conjugated phalloidine (1:200) for 20 min following by Hoechst 33342 (1:1000) for 10 min. Images of bone sections were visualized using confocal microscopy (Leica SP8, Leica) or a fluorescence microscope IX81 (Olympus) equipped with a CCD camera.

### *In vitro* osteoclastogenesis assays

For TRAP stain, induced cells were fixed in 4% paraformaldehyde for 10 min and then stained with TRAP staining solution according to the manufacturers’ instructions (Wako). The images were captured by a fluorescence microscope IX81 (Olympus).

Relative TRAP activity was tested by using murine TRAP ELISA Kit (Jingmei) according to the manufacturers’ instructions. Briefly, 10 μl of sample per well was added after diluted with 40 ul diluent. Then 100 ul of conjugate reagent per well was added and incubated in 37°C for 60 min. After washing for five times, total 100 ul of color developing agent was added and incubated in 37°C for 15 min. The reaction was terminated by adding stop buffer. The absorbancy at 450 nm was measured by colorimetric analysis.

### qRT-PCR analysis

Total RNA isolation was performed using TRIzol reagent and reversely transcribed into cDNA using the RevertAid First Strand cDNA Synthesis kit (Thermo Fisher Scientific) following the manufacturer's protocol. The mRNA expression levels were normalized to GAPDH. Reactions were performed under following cycling conditions: denaturation at 95°C, followed by 40 cycles of denaturation at 95°C for 15 s, then annealing at 60 °C for 1 min. Relative target gene expression was calculated using the 2-ΔΔCq method. Specific primer sequences used for PCR are listed as below: CCL3 (RE: AGGAAAATGACACCTGGCTGG; FW: ACTGCCTGCTGCTTCTCCTACA), GAPDH (RE: TGTAGACCATGTAGTTGAGGTCA; FW: AGGTCGGTGTGAACGGATTTG).

### Western blots

Cell extracts (50 μg of protein) was loaded onto SDS-PAGE gels and blotted on polyvinylidene fluori (PVDF) membranes (Bio-Rad Laboratories). Then membranes were blocked with 5% BSA diluted in PBS. Membranes were then incubated overnight at 4 °C in PBS containing 5% BSA with primary antibody. The blots were then incubated with secondary antibodies labeled with HRP. Signal was detected using a scanner (ChemiDoc Touch Imaging System). The primary and secondary antibodies (all purchased from Cell Signaling Technology) used were as below: Rabbit antimouse phospho-p44/42 MAPK antibody (p-Erk1/2), Rabbit antimouse p44/42 MAPK antibody (Erk1/2), Rabbit antimouse phospho-STAT3, Rabbit antimouse STAT3, Rabbit antimouse phospho-CREB, Rabbit antimouse CREB, Rabbit antimouse GAPDH.

### Statistical analysis

Results are showed as means ± SE as required. Student's *t*-test was used in comparison of two groups. One-way analysis of variance (ANOVA) was used for comparison among three or more groups. Statistical significance was considered at *P*<0.05. *P<0.05, **P<0.01, ***P<0.001. All experiments were repeated at least three times.

## Results

### CRC cells lead to activation of osteoclasts and osteolysis *in vivo*

**Figure 1:**
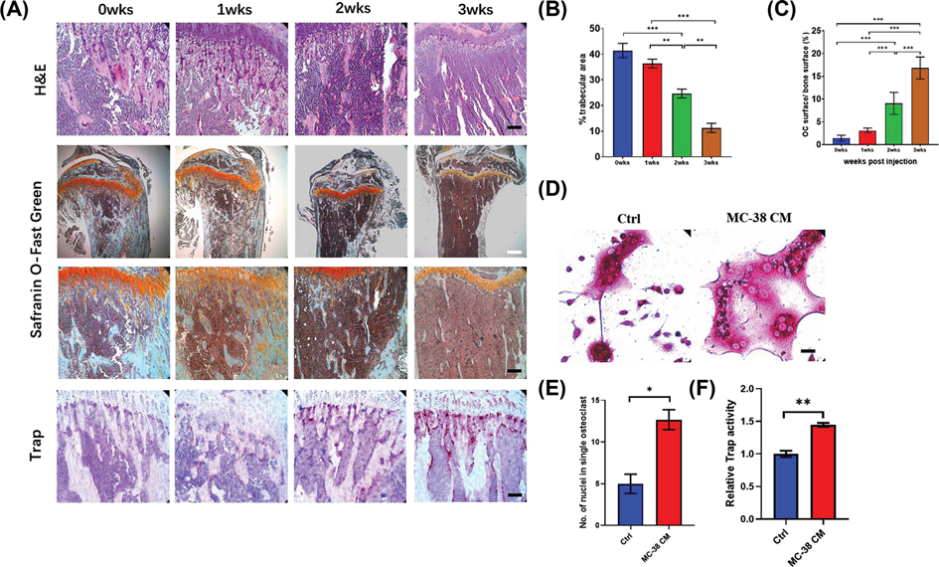
CRC cells lead to activation of osteoclasts and osteolysis *in vivo* (**A**) HE staining and Safranin O-Fast Green staining showing the trabecular area and Trap analysis showing the number of Trap (+) cells in bone metastasis of CRC over time after intratibially injection of MC-38 cells (black scale bar = 100 μm and white scale bar = 250 μm). (**B**) Quantification of trabecular area from Safranin O-Fast green staining in (**A**). (**C**) Quantification of osteoclast number in (**A**). (**D**) Trap staining revealing the effect of MC-38 CM on osteoclastogenesis of BMMs following stimulation with RANKL (50 ng/ml) (scale bar = 20 μm). (**E**) Quantification of nuclei number per osteoclast in (**D**). (**F**) Relative Trap activity assay showing the Trap activity in BMMs treated by MC-38 CM plus RANKL. Abbreviations: wks, weeks; Ctrl, control; CM, conditional medium; No., number. *P<0.05, **P<0.01, ***P<0.001.

We intratibially injected MC-38 to establish the bone metastasis model of CRC. Safranin O-Fast green staining and H&E staining showed trabecular area decreased over time (Figure 1A). Quantification analysis showed bone resorption accelerated from 2 weeks post injection (Figure 1B). Consistent with the bone resorption, the ratio between surface of Trap (+) cells and bone surface increased significantly from 2 weeks post injection (Figure 1A,C). These data suggested CRC can promote the progression of osteolytic lesions in bone.

Then we investigated whether CRC cells regulated osteoclastogenesis of BMMs. Interestingly, BMMs stimulated by RANKL and MC-38 CM enhanced the infusion, the number of nuclei in CM treated group significantly more than the number in control group (Figure 1D,E). Consistent with this, Trap activity was also up-regulated in CM treated group (Figure 1F). These results indicated CRC cells may promote osteoclastogenesis through enhancing the BMMs infusion.

### CCL3 is up-regulated during osteoclastogenesis of BMMs treated with secreta from CRC cells

**Figure 2:**
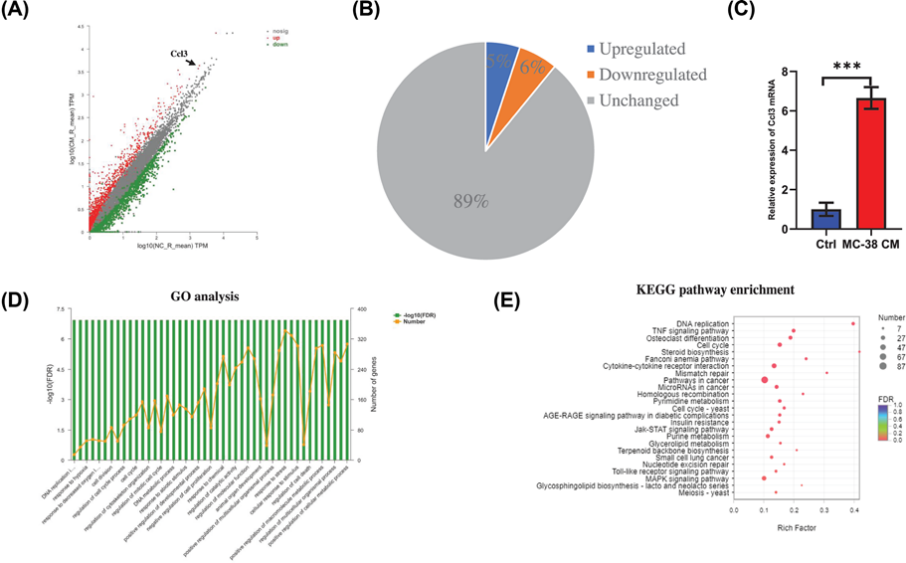
CCL3 is up-regulated during osteoclastogenesis of BMMs treated with secreta from CRC cells (**A**) Scatter plots showing the differentially expressed genes in BMMs during osteoclastogenesis between MC-38 CM treated group and control group. (**B**) Pie chart revealed the percentage of up-regulated, down-regulated, and unchanged genes in MC-38 CM treated group comparing with control group. (**C**) RT-PCR analysis showing the mRNA expression of CCL3 in MC-38 CM treated group and control group. (**D**) GO functional clustering of genes that were differently regulated for biological processes. (**E**) KEGG pathway analysis of different regulated targets in the transcriptome. Abbreviations: Ctrl, control; CM, conditional medium. *P<0.05, **P<0.01, ***P<0.001.

To investigate how CM regulates BMMs during osteoclastogenesis, transcriptomics of BMMs cultured in MC-38 CM as well as RANKL was analyzed. Overall, 1072 genes were up-regulated while 1232 genes were down-regulated in CM treated group comparing with control group (Figure 2A,B). Go analysis showed differentially expressed genes were associated with cytoskeleton organization and cell division (Figure 2D). KEGG pathway analysis revealed that Jak-STAT signaling, MAKP signaling and cytokine-cytokine receptor interaction pathways were highly enriched (Figure 2E). Interestingly, the level of Ccl3 in CM treated group was much higher than the level in control group (Figure 2A). CCL3 was reported to associate with osteoclast formation through regulating the macrophage infusion [12]. Thus, we examined the mRNA level of Ccl3 by using RT-PCR, the result confirmed that the mRNA level of Ccl3 up-regulated after treated with CM (Figure 2C).

### CCL3 mediates osteoclastogenesis of BMMs in CRC microenvironment

**Figure 3:**
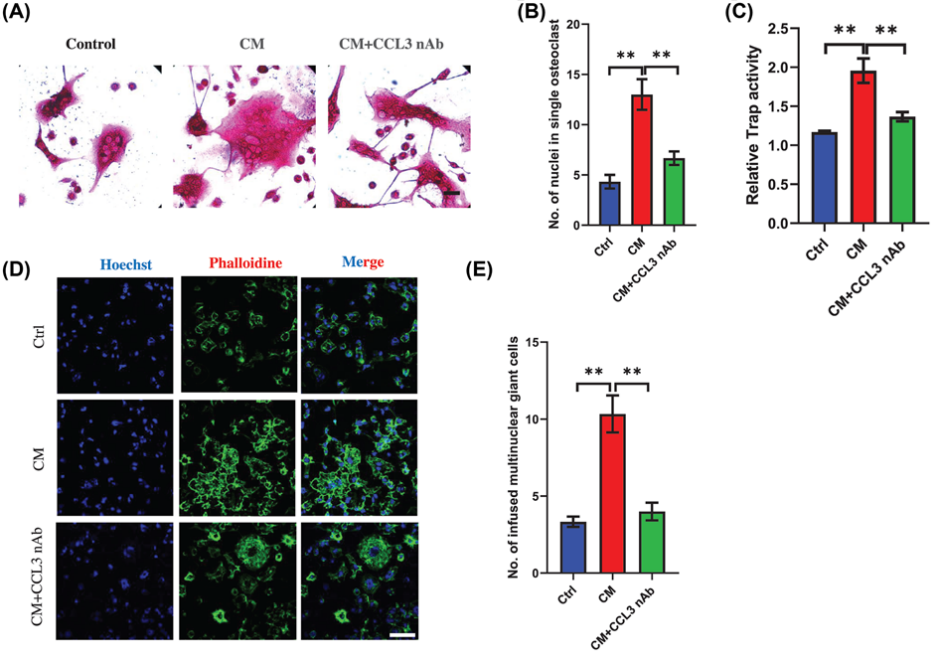
CCL3 mediates osteoclastogenesis of BMMs in CRC microenvironment (**A**) Trap staining showing the osteoclastogenic capacity of BMMs treated by MC-38 CM plus RANKL with/without CCL3 neutralizing antibody. (**B**) Quantification of nuclei number per osteoclast in each group in (**A**). (**C**) Relative Trap activity assay revealing Trap activity in BMMs during osteoclastogenesis in each group in (**A**). (**D**) Immunofluorescence analysis showing the multinuclear giant cell number in BMMs treated with RANKL (Ctrl), MC-38 CM plus RANKL (CM) or MC-38 CM, RANKL, and CCL3 neutralizing antibody (CM+CCL3 nAb) (scale bar = 50 μm). (**E**) Quantification of multinuclear giant cells in each group in (**D**). Abbreviations: nAb, neutralizing antibody; CM, conditional medium; Ctrl, control; No., number. *P<0.05, **P<0.01, ***P<0.001.

Next, we investigated the function of CCL3 in osteoclastogenesis in CRC environment. Trap staining showed the number of nuclei per osteoclast in CM treated group was much more than the number in control group, however, the osteoclast formation stimulated by CM can be significantly prevented after treatment with CCL3 neutralizing antibody (Figure 3A,B). In addition, Trap activity assay also showed inhibition of CCL3 can reverse the enhanced Trap activity stimulated by CM (Figure 3C). To further confirm this result, we analyzed the number of multinuclear giant cells, CM can remarkably stimulate the formation of multinuclear giant cells and blockage of CCL3 can inhibit this process (Figure 3D,E). Together, these results demonstrated that CCL3 played an important role in osteoclast infusion mediated by CRC.

### Activation of EGF/ERK/CREB pathway up-regulates the expression of CCL3 in BMMs during osteoclastogenic induction

**Figure 4:**
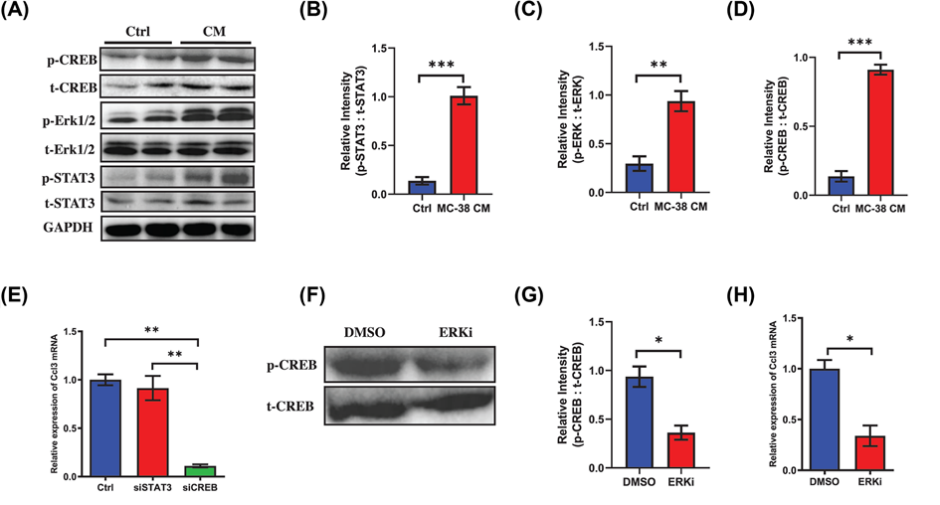
Activation of EGF/ERK/CREB pathway up-regulates the expression of CCL3 in BMMs during osteoclastogenic induction (**A**) Western blot analysis showing the activation of CREB, ERK1/2 and STAT3 in BMMs in MC-38 CM treated group or control group during osteoclastogenesis. (**B–D**) Quantification of relative intensity between phosphorylated CREB and total CREB, phosphorylated ERK1/2 and total ERK1/2, phosphorylated STAT3 and total STAT3, respectively. (**E**) RT-PCR showing mRNA level of CCL3 in BMMs stimulated by MC-38 CM and RANKL and interfered with siSTAT3 or siCREB, respectively. (**F**) Western blot analysis revealing the activation of CREB in BMMs stimulated by MC-38 CM plus RANKL in ERK inhibitor treated group and control group. (**G**) Quantification of relative intensity between phosphorylated CREB and total CREB in (**F**). (**H**) RT-PCR showing mRNA level of CCL3 in BMMs stimulated by MC-38 CM and RANKL in ERK inhibitor treated group or control group. Abbreviations: p, phosphorylated; ERKi, ERK inhibitor; Ctrl, control; CM, conditional medium. *P<0.05, **P<0.01, ***P<0.001.

Previous reports and bioinformatic analysis (www.genecards.org) showed ERK/CREB and STAT3 could be the potential upstream of CCL3 [13,14], thus we examined the activated protein levels of these two pathways in BMMs. As expected, the levels of phosphorylated-STAT3, phosphorylated-ERK1/2 and phosphorylated-CREB were all up-regulated in CM treated group (Figure 4A–D). To find out which one could be the predominant pathway that regulating the expression of CCL3, siRNAs were transfected into BMMs to interfere the STAT3 and CREB. Interestingly, the mRNA level of Ccl3 down-regulated remarkably after down-regulation of CREB while there was little effect after inhibiting the expression of STAT3 (Figure 4E). This result showed CREB was the transcriptional factor of CCL3 in BMMs stimulated by CRC. Then we further explored the effect of ERK signaling on the activation of CREB, as expected, the phosphorylated-CREB was significantly prevented in BMMs treated by ERK antagonist (Figure 4F,G). Moreover, the expression of CCL3 also down-regulated significantly in ERK antagonist treated group (Figure 4H). These findings revealed that the expression of CCL3 in BMMs was regulated by ERK/CREB pathway.

**Figure 5:**
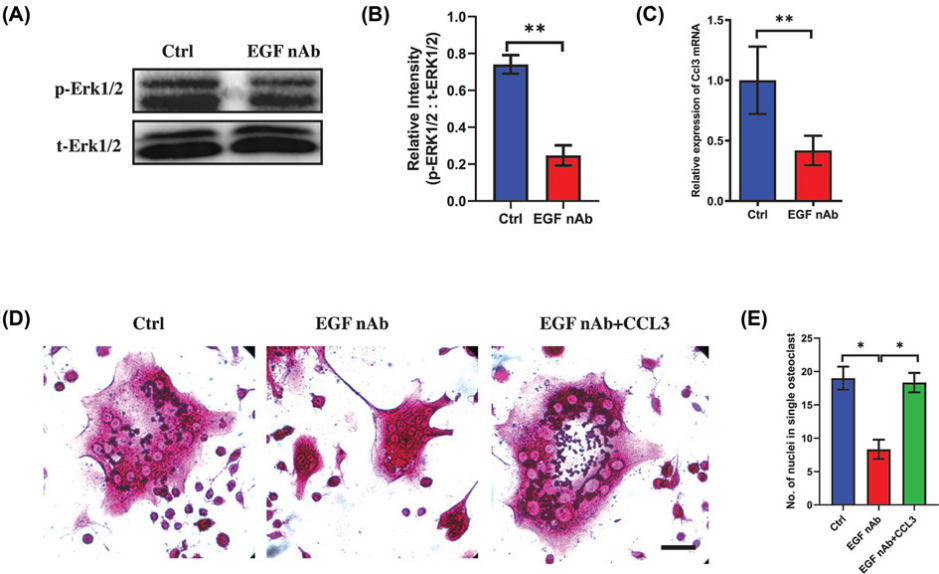
CRC cells derived EGF activates the ERK pathway in BMMs during osteoclastogenesis (**A**) Western blot analysis showing the activation of ERK1/2 in BMMs stimulated by MC-38 CM plus RANKL in EGF neutralizing antibody treated group and control group. (**B**) Quantification of relative intensity between phosphorylated ERK1/2 and total ERK1/2 in (**A**). (**C**) RT-PCR showing mRNA level of CCL3 in BMMs stimulated by MC-38 CM and RANKL in EGF neutralizing antibody treated group and control group. (**D**) Trap staining showing the osteoclastogenic capacity of BMMs treated by MC-38 CM plus RANKL with/without EGF neutralizing antibody and recombinant CCL3 protein (scale bar = 20 μm). (**E**) Quantification of multinuclear giant cells in each group in (**D**). Abbreviations: p, phosphorylated; nAb, neutralizing antibody; Ctrl, control; No., number. *P<0.05, **P<0.01, ***P<0.001.

Recent one study indicated the EGF can be efficiently produced in MC-38 cells and EGF could activate the ERK pathway [15,16], thus we next explored whether MC-38-derived EGF contributed to the overexpression of CCL3 through ERK pathway. Interestingly, the activation of ERK1/2 was prevented in BMMs cultured in CM after treatment with EGF neutralizing antibody (Figure 5A,B). Moreover, the mRNA level of Ccl3 also down-regulated in EGF neutralizing antibody treated group (Figure 5C), indicating blockage of EGF in CM can inactive ERK1/2 pathway and inhibit the overexpression of CCL3. To investigate whether EGF can affect the osteoclastogenesis through CCL3, BMMs cultured in CM plus RANKL was treated by EGF neutralizing antibody with/without recombinant CCL3 protein. Trap staining showed the BMM infusion was prevented in EGF neutralizing antibody treated group and this can be reversed after treated by CCL3 (Figure 5D,E). Altogether, these findings revealed that EGF/ERK/CREB pathway regulated the overexpression of CCL3 in BMMs in CRC microenvironment.

### Blockage of EGF or CCL3 can efficiently attenuate the bone resorption in bone metastasis of CRC

**Figure 6:**
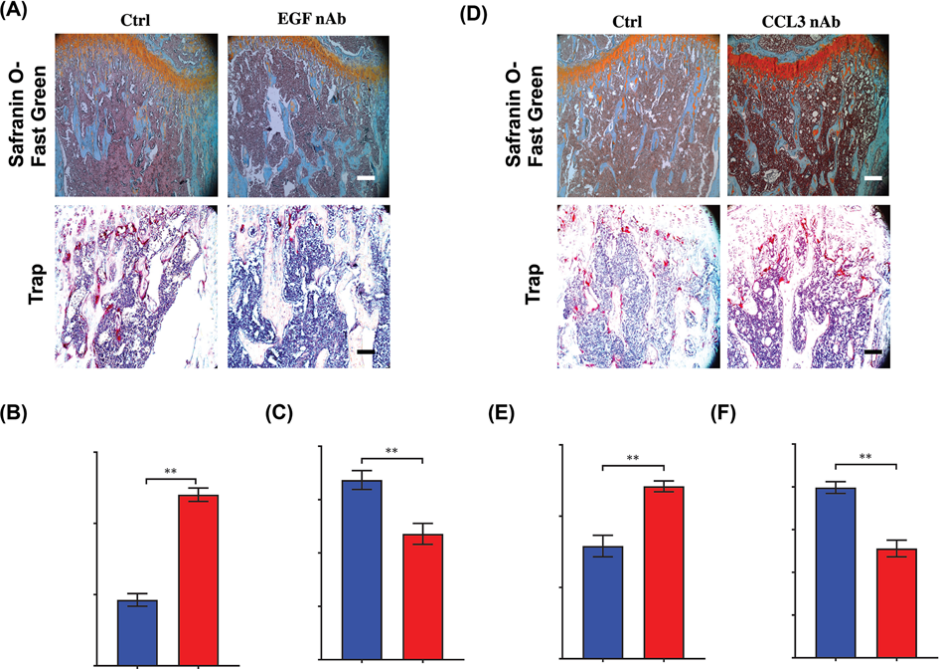
Blockage of EGF or CCL3 can efficiently attenuate the bone resorption in bone metastasis of CRC (**A**) Safranin O-Fast Green staining showing the trabecular area and Trap analysis showing the number of Trap (+) cells in bone metastasis of CRC after treated with EGF neutralizing antibody at 21 days after intratibially injection of MC-38 cells (scale bar = 100 μm). (**B**) Quantification of trabecular area from Safranin O-Fast green staining in (**A**). (**C**) Quantification of osteoclast number in (**A**). (**D**) Safranin O-Fast Green staining showing the trabecular area and Trap analysis showing the number of Trap (+) cells in bone metastasis of CRC after treated with CCL3 neutralizing antibody at 21 days after intratibially injection of MC-38 cells (scale bar = 100 μm). (**E**) Quantification of trabecular area from Safranin O-Fast green staining in (**D**). (**F**) Quantification of osteoclast number in (**D**). Abbreviations: nAb, neutralizing antibody; Ctrl, control; No., number. *P<0.05, **P<0.01, ***P<0.001.

Considering the important role of EGF and CCL3 in osteoclastogenesis in CRC, we next explored the effects of blockage of EGF or CCL3 on bone metastasis of CRC. After treatment of EGF or CCL3 neutralizing antibody, the trabecular area was preserved at 14 days after injection of MC-38 cells (Figure 6A,B,D,E). Consistent with these results, Trap staining showed the relative area of osteoclasts comparing with bone area was reduced after treatment with EGF or CCL3 neutralizing antibody (Figure 6A,C,D,F). These findings demonstrated that EGF and CCL3 were critical in osteolytic formation in bone metastasis of CRC, blockage of these two factors can prevent the CRC-mediated bone loss.

## Discussion

Although clinical and experimental evidence indicates CRC can cause osteolytic lesions in bone metastasis, it remains elusive how osteoclastogenesis is activated in CRC and how CRC cells interact with osteoclasts or the precursors. In our research, we indicate the importance of CCL3 in osteoclastogenesis in bone metastasis of CRC. In addition, we also explore a novel mechanism that CCL3 production in BMMs is regulated by CRC cells derived EGF.

During the osteoclastogenic induction, macrophage infusion is critical to the osteoclast formation. BMMs can commit to osteoclast fate after stimulated by M-CSF and RANKL, then they will form the multinuclear giant cells. Previous report revealed that the serum level of CCL3 was significantly with the increased tartrate-resistant acid phosphatase and the severity of osteoporosis in females [17]. Moreover, the overexpression of CCL3 also positively associated with hypercalcemia [18], implying that CCL3 may regulate the bone resorption. Recently, one study revealed inhibiting CCL3 can abrogate osteoclast precursor cell infusion in human osteoclast cultures and attenuate bone erosion in arthritis [12]. However, it was unknown whether CCL3 participated during bone metastasis in tumorigenic environment. In our CRC bone metastatic model, we found that CCL3 was remarkably overexpressed in BMMs in CRC environment. Similar with previous studies, we revealed osteoclastogenesis can be enhanced in high level of CCL3 through facilitating the BMMs cell infusion. Blockage of CCL3 can efficiently prevent the bone resorption and inhibit the increased osteoclasts *in vivo*. Our findings highlighted the key role of CCL3 in deteriorating osteolysis in bone metastasis of CRC.

Several pathways regulate the CCL3 in varieties of cell types. NF-kappaB signalling promoted the expression of CCL3 in multiple myoloma cells [19]. In addition, ERK pathway was also reported to be upstream of CCL3 in multiple myeloma cells [14,20,21]. Bioinformatic analysis showed STAT3 could be the transcriptional factor of CCL3. To find out which pathway may dominantly regulate the production of CCL3 in BMMs in CRC environment, we analyzed obviously changed pathways in transcriptomic profiling and found both of MAPK pathway and JAK-STAT pathway significantly changed. However, further investigation showed only ERK/CREB, but not STAT3, regulated the expression of CCL3 in BMMs cultured in MC-38 CM. Numerous studies revealed the positive role of ERK pathway in progression of CRC. The aberrant activation of ERK1/2 facilitated the proliferation and immigration of CRC cells and also promoted the metastasis [22–26]. Meanwhile, ERK pathway was well known to regulate the osteoclastogenesis, many signals can enhance the osteoclast formation through activation of ERK pathway [27–31], but downstream of ERK in regulating osteoclastogenic induction was unclear. Our finding revealed that CCL3 was at least one of downstream effectors of ERK that regulating osteoclastogenesis.

As MC-38 expresses high level of EGF and EGF is a predominant activator of ERK pathway in cancers [15,32,33], we investigated the function of EGF on regulating activation of ERK pathway and found blockage of EGF in MC-38 CM efficiently prevented the activation of ERK and inhibited the expression of CCL3, indicating CRC cells derived EGF at least partially activated ERK/CREB/CCL3 signaling in BMMs. In addition, blockage of EGF *in vivo* can remarkably attenuate the bone resportion and osteoclast formation in bone metastasis of CRC, demonstrating that EGF is an important regulator in promoting bone destruction in CRC. It was reported that tumor cells can promote the osteoclast formation through secreting a series of osteoclastogenic cytokines via a RANKL dependent or independent ways, including IL-11, VEGF, CTGF, PTHrP [34], here we found CRC cells can regulate the osteoclastogenesis via indirectly regulating the production of CCL3 through secreting EGF.

In conclusion, we demonstrated that secreta from CRC cells can promote the osteoclastogenesis both *in vitro* and *in vivo*. CCL3 is a key regulator during this process. In addition, ERK/CREB pathway can be activated in BMMs by CRC cells derived EGF and promote the production of CCL3. Targeting EGF or CCL3 can both efficiently reduce the bone loss in bone metastasis of CRC. Our findings suggested EGF and CCL3 could be a potential target in treatment of bone metastasis of CRC.

## Abbreviations

BMM: bone marrow-derived monocyte/macrophage
CCL3: C-C motif chemokine ligand 3
CM: conditioned medium
CRC: colorectal cancer
EGF: epidermal growth factor
FBS: fetal bovine serum
GO: Gene Ontology
MEM: α-minimal essential medium
PFA: paraformaldehyde
PVDF: polyvinylidene fluori
RSEM: RNA-Seq by Expectation-Maximization
TRAP: tartrate resistant acid phosphatase
